# A New Approach for Inversion of Large Random Matrices in Massive MIMO Systems

**DOI:** 10.1371/journal.pone.0094958

**Published:** 2014-04-14

**Authors:** Muhammad Ali Raza Anjum, Muhammad Mansoor Ahmed

**Affiliations:** Department of Electronic Engineering, Mohammad Ali Jinnah University, Islamabad, Pakistan; National Institute of Environmental and Health Sciences, United States of America

## Abstract

We report a novel approach for inversion of large random matrices in massive Multiple-Input Multiple Output (MIMO) systems. It is based on the concept of inverse vectors in which an inverse vector is defined for each column of the principal matrix. Such an inverse vector has to satisfy two constraints. Firstly, it has to be in the null-space of all the remaining columns. We call it the null-space problem. Secondly, it has to form a projection of value equal to one in the direction of selected column. We term it as the normalization problem. The process essentially decomposes the inversion problem and distributes it over columns. Each column can be thought of as a node in the network or a particle in a swarm seeking its own solution, the inverse vector, which lightens the computational load on it. Another benefit of this approach is its applicability to all three cases pertaining to a linear system: the fully-determined, the over-determined, and the under-determined case. It eliminates the need of forming the generalized inverse for the last two cases by providing a new way to solve the least squares problem and the Moore and Penrose's pseudoinverse problem. The approach makes no assumption regarding the size, structure or sparsity of the matrix. This makes it fully applicable to much in vogue large random matrices arising in massive MIMO systems. Also, the null-space problem opens the door for a plethora of methods available in literature for null-space computation to enter the realm of matrix inversion. There is even a flexibility of finding an exact or approximate inverse depending on the null-space method employed. We employ the Householder's null-space method for exact solution and present a complete exposition of the new approach. A detailed comparison with well-established matrix inversion methods in literature is also given.

## Introduction

Multiple-Input Multiple-Output (MIMO) systems form a well established area of wireless communications [1]. A significant increase in interest in MIMO systems has been witnessed in the last few years due to the advent of massive MIMO systems [2], [3]. In these systems, more degrees of freedom in terms of data rate and link reliability are available due to the increase in the number of transmit and receive antennas. These advantages become even more impressive in multi-user scenario when there is a possibility to transmit to several users simultaneously [2]. However, there is still a challenge of low complexity detection techniques for the practical realization of such systems [4]. Many high performance detection methods for massive MIMO systems require an unconstrained solution to a linear estimation problem [5]. Linear estimation requires the inversion of channel matrix which, in such systems, can be problematic because of its potentially large size. For example, matrices of size 40*40 and above have recently been reported in literature as massive [2].

Therefore, there is a need to find solutions that do not require outright inversion. Various methods in this regard have been proposed in literature: Cayley-Hamilton method, Neumann series method, QR method, random matrix methods, LSMR, LSQR, Kaczmarz method and the ones based on polynomial and truncated polynomial filters [6]–[19]. These methods still require a lot of computational effort and some of them have even proven to be more complex than the outright inversion [2], [6]. While it is possible that some may perform better than other, it is not always possible to have a fair comparison among them since these methods follow independent approaches and are dependent on different sets of parameters.

Keeping that in view, our objective in this paper is to develop an alternate method to find the inverse of the channel matrix. While addressing this problem, we dispense with three fundamental assumptions that are generally implied in the traditional methods. We endeavor to do so in order to provide more room for thought. First, no assumption has been made about the structure of the channel matrix. Secondly, the matrix can be purely random instead of being deterministic. Finally, we do not assume that it is sparse [20]. Keeping this in view, we report a novel method that not only finds the inverse of a channel matrix but also brings a new perspective to the inversion process itself. The proposed method is a comprehensive one and is fully applicable to all three matrix inversion cases pertaining to a linear system. We also provide its detailed comparison with well-known matrix inversion methods available in literature for the sake of analysis and, hence, understanding.

Rest of the article is organized as follows. To begin with, the subsequent section lays out basic system model and the essential nomenclature. The third section presents the basic idea behind the proposed method. The inversion problem is solved according to the proposed method using Householder's null-space method in the fourth section. In fifth section, solution to three fundamental cases of linear systems is presented. Proposed method is then compared with the QR decomposition (QRD) method, Least Squares (LS) method, and Moore and Penrose's pseudoinverse method in sections six, seven, and eight respectively. A complete algorithm for step by step computation of the inverse matrix according to the proposed method is outlined in section nine. Simulation results demonstrating the speed and accuracy of the proposed method in comparison to the state of the art methods available in literature are presented in section ten. Finally, the article concludes with a brief discussion in section eleven.

## System Model

A MIMO system is represented by a system of linear equations [21].
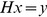
(1)



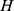
 is the channel matrix of dimension 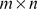, 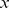 is the transmitted vector of dimension 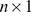, and 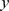 is the received vector of dimension 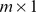. Entries of 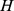 matrix are complex-valued independent and identically distributed Gaussian random variables of zero mean and unity variance [6]. 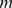 and 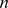 are the number of transmit and receive antennas respectively. If 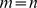, the number of equations is equal to the number of unknowns and the system is fully-determined. If 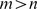, the number of equations is greater than the number of unknowns and the system is over-determined. If 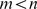, the number of equations is less than the number of unknowns and the system is under-determined.

### Basic Idea

We begin with the assumption that 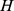 has a full-rank. Re-writing Eq. (1) as:
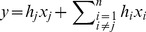
(2)


First term on the right hand side of Eq. (2) denotes 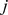-th column of matrix 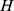.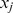 is the associated unknown. The second term represents the remaining columns of 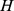 with their respective unknowns. This term can be separately written in matrix form by defining a new column reduced matrix 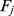 and a symbol reduced vector 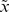. 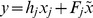
(3)



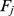
 has dimensions of 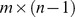. Taking the dot product of Eq. (3) with an arbitrary column vector 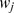 of dimension 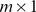,
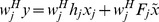
(4)


To evaluate the *j*-th unknown 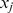 from Eq. (4),
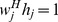
(5)

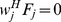
(6)


Eqs. (5) and (6) define the 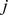-th row of the inverse matrix. It should have a dot product of one with the 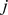-th column and zero with all the remaining columns. The same is also true for all the other rows of the inverse matrix. Once we are able to solve Eqs. (5) and (6) for 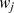, all rows of inverse matrix can be computed one by one in the same fashion and a complete inverse matrix can be built. Eq. (5) essentially restricts the length of the null-space solution produced by Eq. (6) and serves as a constraint for Eq. (6). Therefore, we proceed by solving the Eq. (6) first. It can be solved by a variety of methods available in literature of for computing the null-space of a matrix. We propose to solve it by Householder's null-space method because it is stable and provides an exact solution.

### Solution Using Householder's Null-Space Method

Householder's method has been traditionally viewed as a means to achieve the QR decomposition (QRD). It performs a series of orthogonal transformations on an arbitrary matrix to convert it to an upper triangular matrix. These transformations can be performed either by Given's rotation matrices or Householder's reflection matrices. A reflection matrix can perform the job of many rotation matrices at once. Therefore, reflection matrices are more desirable in our case. We proceed by performing a series of orthogonal transformations on the matrix 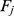 using the reflection matrices to convert it to an upper triangular matrix 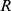. 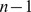 reflection matrices will be required for that purpose because 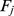 has 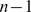 columns.
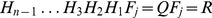
(7)



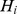
's represent the Householder's reflection matrices. They are square, symmetric and orthogonal with each one having the dimensions of 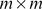. 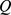 has also the same dimensions. But the dimensions of 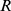 are 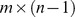. This is because the first 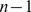 rows of 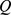 generate an 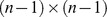 upper triangular portion in 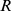. Remaining 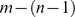 rows in 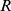 are zero. These rows are produced by last 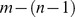 rows of 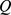.
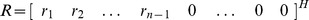


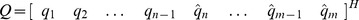
(8)

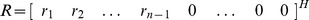
(9)



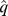
's mark the rows that produce zeros in 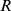 matrix. Substituting Eqs. (8) and (9) in Eq. (7), 
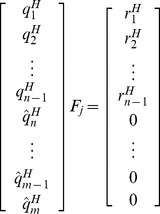
(10)


Concentrating on 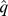
's only in Eq. (10),
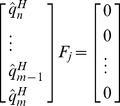
(11)



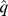
's form the basis for the left null-space of 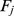 and, hence, solve Eq (6). Only equation that remains to be solved now is Eq. (5). In order to do so, we would need to consider the three fundamental cases of linear systems.

## Three Fundamental Cases

In this section, we take up the three fundamental cases of linear systems and solve them one by one.

### 1. The Fully-Determined Case

In the fully-determined case, there is only one vector in the left null-space of 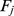, i.e., 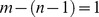 given that 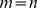. The solution in Eq. (11) is unique.
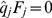
(12)


The only remaining task is to rescale 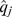 according to Eq. (5). 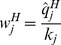
(13)


Whereas,
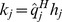
(14)



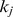
 is the rescaling factor for the *j*-th row of inverse matrix 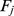. By iterating 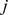 for all columns of 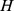 matrix, complete inverse matrix 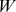 can be built.
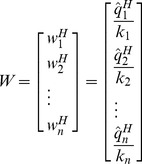
(15)such that,
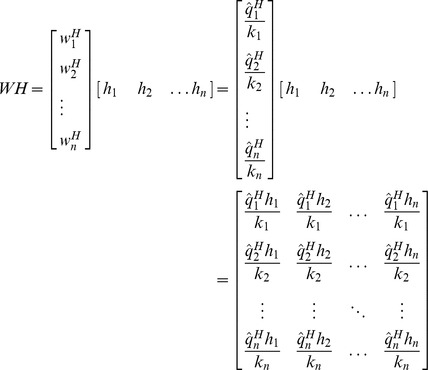


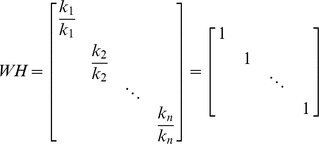



The identity matrix will have the dimensions of 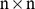.

### 2. The Over-Determined Case

In the over-determined case, the matrix 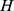 will already have a right null-space due to a greater number of rows than the columns. The removal of extra column to form 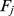 will populate the null-space even further and there will be 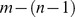 vectors in the left null-space of 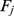. Hence, the solution to the Eq. (6) will not be unique.
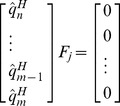
(16)


All the vectors from 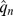 to 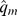 solve Eq. (6). An obvious question would be which one to choose. At this point, we adjourn this question to Section VIII where it would be amply addressed. For now, any one of them can be selected. The next step would be to rescale it according to Eq. (13). By repeating the same procedure for all columns of 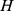, a complete inverse matrix can be built.

### 3. The Under-Determined Case

In the under-determined case, 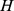 matrix will have right null-space due to a greater number of columns than the rows. In order to be consistent with the earlier work, we can transpose it and move the null-space to left. The inverse matrix can then be computed in the manner described in subsection (b). Only a transposition of inverse matrix is required afterwards to revert to the original case.

### Comparison with QR Decomposition

Traditional methods employing QR decomposition take a linear system of the form,
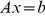
(17)into,
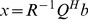
(18)


by factorizing 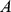 in Eq. (17) into an orthogonal matrix 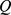 and an upper-triangular matrix 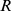 [22]. There are two famous ways for achieving QR decomposition: Gram-Schmidt QR and Householder's QR [22]. Traditionally, Gram-Schmidt method has been used for that purpose. Objective was to convert an 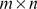 matrix 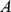 into an 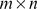 orthogonal matrix 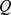. Matrix 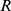 connects 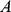 to 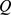.
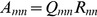
(19)


Householder's method takes a shift in perspective. 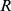 is targeted instead of 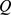. Being an upper triangular matrix, 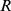 is much more suitable for back substitution. 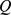 serves as the connecting matrix. 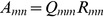
(20)


The 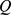 in Eq. (20) has extra 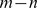 columns than the one obtained by Gram-Schmidt method. First 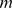 columns serve as the orthonormal basis for the column space of 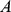. Remaining 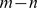 columns form the basis for the left null-space of 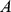 because 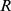 can only be made triangular upto first 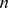 rows. [22]. 
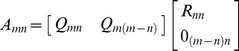
(21)


In the proposed method, Househoder's method is employed to find the null space basis 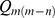 of 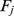. Also, there is a difference in the orientation of 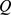 in Eq. (8) which leads to,
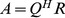
(22)


instead of 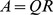. We take 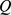 as such because its last 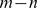 rows are responsible for the zero last 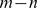 rows of 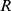. Orthonormal column space of 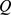 in Eq. (20) is now an orthonormal row space in Eq. (22).

### Comparison with Least Squares

When 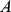 has fewer columns than the rows, the system becomes over-determined. There are more equations than the unknowns. The solution is then obtained by Least Squares (LS).
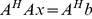
(23)


QR decomposition is a popular method to solve LS problems and when applied to Eq. (23) will result in Eq. (18).

We have not approached the over-determined case using LS. We have taken it column by column. A dearth of columns in an over-determined matrix signals an apriori presence of the left null-space. Removal of one more column will populate it further. This will bring us to select, out of this abundance of null-space vectors, the one which has the highest value of 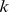 depicted in Eq. (14). It needs to be so because the selected vector will then be divided by 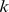 according to Eq. (13). Smaller values of 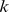 can inflate the magnitude of the null-space vector after division. In the worst case of 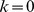, there will be a division by zero. Therefore, out of many null-space vectors obtained by Householder's null-space method, the best would be the one which has the highest value of 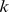 for the specific column vector. The term best can be pushed even further. In ideal case, a value of one for 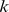 would imply no division at all. But the caveat is to avoid smaller values of 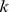 in order to prevent instability.

### Comparison with Moore and Penrose's Pseudoinverse

Now we turn to the last case, the case when the matrix 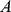 is singular. A solution in this case is sought by forming the Moore and Penrose's pseudoinverse. Not an exact inverse though, the idea is to find the shortest vector that solves the LS equation. The term shortest implies a vector that has no null-space component. One way to do is to use Singular Value Decomposition (SVD) [20]. SVD decomposes the matrix 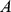 into two square, orthonormal matrices 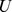 and 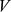. These matrices contain the basis for the column and row space of 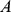 respectively. 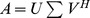
(24)


The third matrix 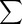 is a diagonal matrix that contains the singular values. Some of the singular values in 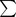 will be zero for this case due to the presence of dependent columns in 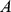. Columns of 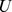 and 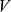 for the corresponding zero singular values will represent the left and right null-spaces of 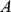. Pseudoinverse is formed by leaving the zero singular values unchanged while inverting the others. This gives the shortest solution in row space.

At this point, a 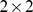 matrix with all entries equal to one would serve best to explain our approach. Once the first column is removed, Eq. (6) will seek a solution in the left null-space of the remaining columns. Eq. (5) will dictate this solution to have a projection of value equal to one with the removed column. This is impossible because both the removed column and the remaining column are exactly the same. A vector cannot be orthogonal and parallel to itself at the same time. In our opinion, this is precisely the reason for non-invertibility of a matrix rather than the traditional zero-determinant view. The only unique vector is the vector left in the column space of 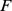, the only remaining column. Therefore, it is rescaled with itself to satisfy Eq. (13). This is exactly the solution obtained by applying the SVD to above problem.

## Algorithm

We summarize the steps discussed in the form of a complete algorithm as follows.

1. If 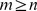, continue to step 2, otherwise transpose the matrix 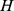 before moving to step 2.
2. Locate the *j*-th column whose inverse vector is to be determined.
3. Remove that column from 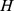 matrix to form a new matrix 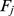.
4. Decompose 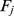 to form 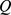 and 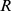 matrices according to Eq. (7).
5. Discard the 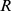 matrix and the first 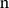 rows of 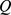 matrix.
6. Remaining 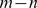 rows will constitute null-space of 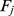. In case 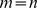, there will be only one row and, hence, one candidate for the inverse channel vector. In case 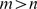, all of the remaining 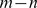 rows satisfy the criteria in Eq. (6). In this case, pick any one at random.
7. Normalize the selected vector according to Eqs. (13) and (14) to form the inverse vector 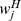.
8. Go back to step 2 and repeat the same procedure for the remaining columns of 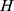 matrix.
9. Stack the inverse channel vectors on top of each other in the manner described in Eq. (15) to form the complete inverse matrix 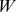.
10. For the case when 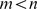, transposition of 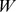 formed in step 9 is necessary to form the right handed inverse.

A user-friendly code of this algorithm is provided in Code S1.

## Simulation Results

We now present the simulation results of the algorithm. The components of the channel matrix are chosen to be independent and identically distributed (IID) circularly symmetric Gaussian random variables with a zero mean and unity variance. 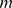 is selected to be equal to 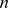 as this refers to multi-user case in M-MIMO systems because both the number of transmitting and receiving antennas become very large. For example, a typical matrix size can be 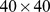 and above [2]. Also when 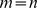, an exact solution is possible and the residue and hence the error in the estimate can be zero.

For simulation purpose, various matrix sizes have been selected and the results obtained in terms of the norm of residue, norm of the error in estimate and the computational time taken are displayed in Table 1 against the state of the art algorithms available in literature: LSMR, LSQR, and Kaczmarz method. Since the proposed method achieves zero error/residue norms, its respective columns are not displayed in the table. Codes required for simulation of LSQR and LSMR algorithms have been downloaded from the website of Stanford University's System Optimization Laboratory and are used as is.

**Table 1:** Comparison of the proposed algorithm with the state of the art methods available in literature.

M = N	Proposed	LSMR			LSQR			Kaczmarz 100 iterations		
	time	||r||	||e||	time	||r||	||e||	time	||r||	||e||	time
N = 20	0	1.6547	17.9982	0.0625	0.0511	0.0167	0.0781	2.6355	21.7263	0.0469
N = 40	0.0156	0.0795	0.2899	0.0313	0.6181	2.3153	0.0938	0.4677	1.2842	0.1406
N = 60	0.0313	1.2373	7.7615	0.0469	2.4645	11.5742	0.0625	2.9948	10.7212	0.2969
N = 80	0.0625	0.4229	2.4126	0.0469	1.0575	4.8856	0.0625	1.3186	5.6604	0.3906
N = 100	0.1250	0.6446	5.5752	0.0625	2.3165	9.5884	0.0938	2.5353	8.4609	0.5156
N = 120	0.2500	1.2121	38.2089	0.0469	3.0198	39.6965	0.0625	3.2040	39.9166	0.7656
N = 140	0.4375	1.4229	13.3729	0.0781	3.0258	18.4997	0.0625	4.1424	18.8697	1.0625
N = 160	0.7344	0.4929	8.3269	0.0469	1.7582	9.7641	0.0781	1.5562	9.8529	1.3281
N = 180	1.2031	0.8819	7.1348	0.0625	2.9170	13.5627	0.0781	3.0961	13.4074	1.7031
N = 200	1.7656	0.9897	19.4026	0.0625	3.0598	24.0314	0.0781	4.2552	24.1649	2.0781

Simulation results demonstrate that when LSMR, LSQR, and Kaczmarz method are applied to this problem, these methods suffer from very large residue norms. But that is not all. The norms for the error in estimate are even worse. Kaczmarz method is not only the slowest of all but also has largest error/residue norms. Hence, the estimates become practically useless. On the other hand, the proposed method achieves perfectly zero error/residue norms in moderate time. Therefore, the proposed method can be a much better choice in this context due to its better accuracy and speed.

## Conclusion

A novel approach for matrix inversion has been presented in this paper. The matrix was split into a set of column reduced matrices, each with its own inverse channel vector 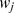. In order to qualify for an inverse channel vector, 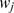 had to satisfy two constraints; 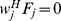 and 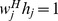. Householder's null-space method was used to solve the first constraint and scaling was used to solve the second one. Inverse matrix was then built from these inverse channel vectors. A detailed comparison with the state of the art methods available in literature was carried out all along to emphasize the novelty of the method.

## Supporting Information

Code S1 **A user-friendly code of the proposed algorithm.**
(ZIP)
